# Association of metabolic syndrome and the risk of bladder cancer: A prospective cohort study

**DOI:** 10.3389/fonc.2022.996440

**Published:** 2022-10-03

**Authors:** Shuo Fang, Yuchen Liu, Huiru Dai, Tianshun Gao, Leli Zeng, Rui Sun, Zilong Zheng, Jinqiu Yuan, Bin Xia, Yihang Pan

**Affiliations:** ^1^ Department of Oncology, The Seventh Affiliated Hospital, Sun Yat-sen University, Shenzhen, China; ^2^ Department of Clinical Oncology, The University of Hong Kong, Hong Kong, Hong Kong SAR, China; ^3^ Big Data Centre, The Seventh Affiliated Hospital, Sun Yat-sen University, Shenzhen, China; ^4^ Centre for Clinical Research and Biostatistics, The Jockey Club School of Public Health and Primary Care, the Chinese University of Hong Kong, Hong Kong, Hong Kong SAR, China; ^5^ Clinical Research Center, The Seventh Affiliated Hospital, Sun Yat-sen University, Shenzhen, China; ^6^ Precision Medicine Center, The Seventh Affiliated Hospital, Sun Yat-sen University, Shenzhen, China

**Keywords:** metabolic syndrome, bladder cancer, independent risk, central obesity, blood glucose

## Abstract

**Background:**

Metabolic syndrome (MetS) and its components have been shown as risk factors for several solid cancers. However, current epidemiological evidence about the relevance of MetS and bladder cancer risk was limited.

**Methods:**

We conducted a prospective cohort study of 476,986 participants with undiagnosed bladder cancer based on the UK Biobank. MetS was defined as the presence of at least three of the five selected indicators: hypertension, central obesity, raised triglyceride, reduced HDL-cholesterol, and raised fasting plasma glucose. Bladder cancer has been identified through contact with the British Cancer Registry (median follow-up time: 6.6 years). We assessed hazard ratio (HR) and 95% confidence interval (CI) through Cox proportional hazard regression after adjusting for demographic and lifestyle factors. Non-linear associations for individual MetS components were assessed by the restricted cubic spline method.

**Results:**

During a follow-up of 3,112,566 person-years, 487 cases of bladder cancer were ascertained. MetS (HR = 1.32, 95% CI = 1.08–1.61), central obesity (HR = 1.39, 95% CI = 1.15–1.68), dyslipidemia for HDL cholesterol (HR = 1.31, 95% CI = 1.04–1.66), and hyperglycemia (HR = 1.44, 95% CI = 1.16–1.79) were associated with elevated risk of bladder cancer. Bladder cancer risk increased with the number of MetS components. In stratified analyses, MetS showed similar effects in bladder cancer independently with sex, age, cigarette and alcohol use, physical activity, and dietary factors. Higher waist circumference, BMI, fasting blood glucose, and glycosylated hemoglobin were independently associated with increased risk of bladder cancer, with no evidence against non-linearity.

**Conclusion:**

MetS might be an independent risk factor for bladder cancer. Our findings highlighted the importance of individualized management of MetS components for preventing bladder cancer.

## Introduction

Bladder cancer is the 10th most common malignancy in the world, with approximately 573,000 new cases and 213,000 deaths in 2020. Additionally, it is more prevalent in Southern Europe, Western Europe, and North America (1). Retrospective studies had reported various risk factors for bladder cancer, such as age, gender, tobacco and alcohol use, arsenic and nitrate in drinking water, traditional medicine like *Aristolochia* plants use, exposure to potential carcinogens at the workplace, and family history (2). Nonetheless, reliable data that track the correlation between physical constitution and bladder cancer are scarce. Therefore, more efforts should be made to identify and implement effective prevention strategies.

Metabolic syndrome (MetS) is a group of metabolic abnormalities including central obesity, hypertension, dyslipidemia, insulin resistance, and subsequent diabetes (3, 4). Previous studies have suggested that MetS was associated with a higher risk of incidence of a major cardiovascular event, thus attracting worldwide attention (5, 6). Many studies have also evaluated the relationship of MetS and the specific symptoms of multiple malignancies such as pancreatic cancer (7), breast cancer (8), renal cancer (9), and prostate cancer (10). To date, their underlying mechanisms have not been fully understood.

Studies have suggested that MetS status was associated with elevated levels of inflammatory factors, reactive oxygen, and serum insulin-like growth factor 1 (IGF-1), which might provide a protumorigenic environment and are risk factors for bladder cancer (11–16). Indeed, experimental studies have shown that reactive oxygen might damage DNA in genes and contribute to tumor cell survival as well as the development of bladder cancer (13, 16). IGF-1 with mitogenic and antiapoptotic activity also stimulates the proliferation of tumor cells. It was suggested that patients with bladder cancer have higher plasma levels of IGF-1 (14, 15). Recent studies have also evaluated the correlation between MetS and bladder cancer. However, inconsistent findings concerning this relationship exist. For example, a meta-analysis conducted by Esposito et al. reported a weak correlation between MetS and bladder cancer in men (RR = 1.10, *p* = 0.013) (17), but Cantiello et al. reported that MetS was not an independent risk factor for bladder cancer (OR 1.006, *p* = 0.983) (18).

Additionally, it has been suggested that some MetS components may have a U-shaped association with the risk of specific cancer, for instance, relationship between waist circumstance and liver cancer (19). However, the non-linear relationships between MetS components and bladder cancer have rarely been investigated.

The UK Biobank is an ongoing multi-center cohort of over 500,000 individuals with detailed measurements of more than 2,000 traits, including sociodemographic assessments, anthropometric measurements, self-reported behavioral outcomes, and clinical diagnoses. The large database of recorded bladder cancer cases provides substantial statistical power to investigate the effects of MetS and its components on bladder cancer, and opens up a new opportunity to explore population-based risk factors for cancer development. Based on this database, we aimed to investigate whether MetS factors, either individually or in the aggregate, are associated with the subsequent occurrence of bladder cancer, and thereafter to examine the non-linear associations for individual MetS components.

## Materials and methods

### Population and data access

The UK Biobank is a well-characterized prospective cohort study that included 502,527 individuals (229,131 men and 273,396 women) aged 37–73 years throughout England, Wales, and Scotland between 2006 and 2010. The UK Biobank is an open-access resource, and the application procedure is available on its website (https://www.ukbiobank.ac.uk/). The UK Biobank has ethical approval from the North West Multi-Centre Research Ethics Committee. All participants had provided written informed consent prior to data collection.

This study obtained approvals from the review committees for the UK Biobank (application number 51671, approved August 2019). Participants who had any cancer (except for non-melanoma skin cancer ICD-10 C44) diagnosis before baseline (*n* = 23,561) were excluded from the analyses. We also excluded participants who lack measurement data for all five components of MetS (*n* = 533), those who are pregnant (*n* = 149), and those who subsequently withdrew from the study (*n* = 1,298). Finally, this analysis involved data from a total of 476,986 participants.

### Measurements

The data collection and quality control methods have been described (20). Waist circumference measurements were collected from participants using a Seca 200-cm tape measure. Body weight was measured by using a calibration scale. The standing height was measured with a tape measure. Body mass index (BMI) was calculated in kg/m^2^. An Omron 705IT automatic digital blood pressure monitor was used to measure the sitting blood pressure two times at 1-min interval and then the systolic blood pressure and diastolic blood pressure readings were averaged. Serum fasting glucose, total cholesterol, high-density lipoprotein cholesterol (HDL-C), and triglycerides were measured using the Beckman Coulter AU5800 analyzer (21). Low-density lipoprotein cholesterol (LDL-C) was measured using a direct Beckman assay. Participant race/ethnicity was assessed using self-reported information. Years of education were calculated based on the standardized International Standard Classification of Education of the United Nations Educational, Scientific, and Cultural Organization (22). The Townsend deprivation index, an area-based proxy measure for socioeconomic status, was also derived for each participant (23). Smoking, alcohol intake status, multivitamin use, mineral supplement, and non-steroidal anti-inflammatory drug use status were all self-reported.

### Assessment of outcome

Incident bladder cancer cases within the UK Biobank cohort were identified through contact with cancer and death registries. Participants contributed person-years from recruitment until first bladder cancer diagnosis (ICD-10 code C67), date of death, or last date of follow-up (31 October 2016 for England and Wales, 31 December 2015 for Scotland), whichever came earliest.

### Definition of MetS and its components

MetS was defined following the International Diabetes Federation (IDF) criteria (24). Participants are classified according to the number of MetS components present at baseline. Participants with more than three components were classified as a MetS: (1) hypertension (systolic blood pressure ≥130 mmHg or diastolic blood pressure ≥85 mmHg); (2) central obesity (waist circumference, male ≥94 cm or female ≥80 cm); (3) raised triglyceride (≥150 mg/dl); (4) reduced HDL-cholesterol (male <40 mg/dl or female <50 mg/dl); and (5) raised fasting plasma glucose (≥100 mg/dl).

### Data analysis

Values were presented as either a mean (standard deviation) or a number (percentage) in descriptive analyses. For covariates with selections of “prefer not to answer” or “do not know”, or with missing covariate data, an “unknown/missing” response category was created.

Cox proportional hazard regression analyses, taking age as the time variable, were performed to calculate the hazard ratio (HR) and 95% confidence interval (95% CI). The proportional hazards assumption was checked using Schoenfeld residuals test. To address potential reverse causation, we performed a lagged analysis of the exposure for 2 years, which could strengthen the temporality and allow a time window for bladder cancer development. To control for confounding factors, our multi-adjusted models adjusted for several covariates associated with bladder cancer risk (25–34). We first stratified the analyses jointly by age, gender, and UK Biobank assessment center to estimate the crude associations of MetS and its components with subsequent bladder cancer risk. To control the potential confounding factors from sociodemographic factors, we additionally adjusted for race (white or other), education (college or university degree, other), index of multiple deprivation (a measure of socioeconomic status), smoking status (never smoked, previous smoker, current smoker), alcohol consumption (never or special occasions only, one to three times a month, one to four times a week, daily or almost daily), physical activity (MET-hours/week, continuous), and portions of fruit and vegetable intake (<5 portions per day, ≥5 portions per day, or unknown/missing) in multivariable adjusted model 2. In the fully adjusted model 3, we additionally adjusted for regular medications [multivitamin use (yes or no), mineral supplement (yes or no), non-steroidal anti-inflammatory drugs (yes or no), and aspirin (yes or no)] and general health indicator variables, including overall health rating (poor, fair, good, excellent, and unknown/missing) and long-standing illness (yes or no).

In addition, we used the restricted cubic method spline to assess the potential non-linearity association of each MetS component with the risk of bladder cancer. In the present study, we used three-knot models with three knots at the 10th, 50th, and 90th percentiles of each MetS component to achieve the best fit, as Harrell et al. recommended. We investigated the non-linearity associations by using a likelihood ratio test comparing the model with only a linear term against the model with linear and cubic spline terms.

We conducted stratified analysis (age, sex, smoker, drinker, physical activity, fruit and vegetable portions, and regular non-steroidal anti-inflammatory drug use) and fitted an interaction term between MetS and each of these factors in our model, to investigate the potential effect modifiers. We also performed a propensity score-matching analysis in the UK Biobank to test the robustness of the results. Briefly, multivariate logistic regression, including the aforementioned covariables, was performed to estimate the propensity score. The MetS participants and non-MetS participants were matched by propensity scores with a 1:2 nearest-neighbor caliper matching method without replacement. We set a caliper of 0.2 SDs of the logit of the propensity score. Standard differences were calculated to evaluate the balance of the baseline covariates between the two groups and a standard difference of 10% or less was considered to indicate good balance. After matching, we fitted Cox regression model to quantify HRs and 95% CIs.

## Results

Among 476,986 participants involved in our study, there were 219,287 men (46.0%) and 257,699 women (54.0%). Demographic and clinical pathologic features of the study population are summarized in **Table 1**. MetS was found in 116,831 participants (24.5%), and was comparable in men (57,700, 49.4%) and women (59,131, 50.6%). The mean age of participants with MetS was 58.6 ± 7.7 years and those with non-MetS was 56.3 ± 8.2 years. Individuals with MetS were more likely to spend less time in physical activity, taking less fruit and vegetable, and consuming more alcohol and cigarette.

**Table 1:** Baseline characteristics.

Characteristics	Non-metabolic syndrome (*N* = 360,155)	Metabolic syndrome (*N* = 116,831)	Overall (*N* = 476,986)
Mean (SD) age, years	56.3 (8.2)	58.6 (7.7)	56.9 (8.1)
Female, N (%)	198,568 (55.1)	59,131 (50.6)	257,699 (54.0)
White, N (%)	340,484 (94.5)	110,401 (94.5)	450,885 (94.5)
College or University degree, N (%)	125,547 (34.9)	27,902 (23.9)	153,449 (32.2)
Mean (SD) Index of multiple deprivation	16.6 (13.6)	19.7 (15.3)	17.4 (14.1)
Mean (SD) waist circumference, cm	85.8 (11.0)	104 (10.8)	90.3 (13.5)
Mean (SD) BMI, kg/m^2^	25.8 (3.7)	32.4 (4.5)	27.4 (4.8)
Median (IQR) physical activity, MET hours/week	31.5 (46.9)	23.2 (41.4)	29.5 (46.0)
Family history of cancer, N (%)	123,612 (34.3)	41,653 (35.7)	165,265 (34.6)
>5 portions of fruit and vegetable per day, N (%)	137,728 (38.2)	41,972 (35.9)	179,700 (37.7)
Mean (SD) TG, mmol/L	1.5 (0.8)	2.4 (1.2)	1.7 (1.0)
Mean (SD) HDL, mmol/L	1.5 (0.4)	1.3 (0.3)	1.5 (0.4)
Mean (SD) LDL, mmol/L	3.6 (0.8)	3.5 (0.9)	3.6 (0.8)
Mean (SD) cholesterol, mmol/L	5.7 (1.1)	5.6 (1.3)	5.7 (1.1)
Mean (SD) fasting glucose, mmol/L	5.0 (0.9)	5.5 (1.7)	5.1 (1.2)
Mean (SD) HbA1c, mmol/L	35.1 (5.0)	39.1 (9.3)	36.1 (6.5)
Mean (SD) SBP, mmHg	138 (19.7)	146 (18.1)	140 (19.7)
Mean (SD) DBP, mmHg	81.0 (10.6)	86.0 (10.3)	82.3 (10.7)
Mean (SD) CRP, mg/L	2.1(4.0)	3.8 (4.9)	2.6 (4.3)
Multivitamin use, N (%)	54,945 (15.3)	16,249 (13.9)	71,194 (14.9)
Intake of mineral supplements, N (%)	78,699 (21.9)	22,942 (19.6)	101,641 (21.3)
Aspirin use, N (%)	39,485 (11.0)	28,726 (24.6)	68,211 (14.3)
Non-aspirin NSAIDs use, N (%)	57,341 (15.9)	20,467 (17.5)	77,808 (16.3)
Smoking status, N (%)			
Current	38,067 (10.6)	12,643 (10.8)	50,710 (10.6)
Previous	116,457 (32.3)	46,677 (40.0)	163,134 (34.2)
Never	205,631 (57.1)	57,511 (49.2)	263,142 (55.2)
Drinking status, N (%)			
Daily or almost daily	76,975 (21.4)	19,680 (16.8)	96,655 (20.3)
1–4 times a week	182,087 (50.6)	52,134 (44.6)	234,221 (49.1)
One to three times a month	38,340 (10.6)	14,762 (12.6)	53,102 (11.1)
Special occasions only/Never	62,753 (17.4)	30,255 (25.9)	93,008 (19.5)
Overall health rating, N (%)			
Excellent	71,291 (19.8)	8,115 (6.9)	79,406 (16.6)
Good	216,217 (60.0)	59,536 (51.0)	275,753 (57.8)
Fair	60,300 (16.7)	37,953 (32.5)	98,253 (20.6)
Poor	10,396 (2.9)	10,269 (8.8)	20,665 (4.3)
Unknown/missing	1,951 (0.5)	958 (0.8)	2,909 (0.6)
Long-standing illness, N (%)	94,870 (26.3)	52,896 (45.3)	147,766 (31.0)

BMI, body mass index; TG, triglyceride; HDL, high-density lipoprotein; LDL, low-density lipoprotein; SBP, systolic blood pressure; DBP, diastolic blood pressure; CRP, C-reactive protein.

We documented 487 cases of bladder cancer during a median of 6.6 years of follow-up. **Table 2** illustrates the risk of bladder cancer in association with MetS and components. Results showed that participants with MetS have 39% elevated risk of bladder cancer than those without (HR = 1.39, 95% CI = 1.15–1.67). These results remained unchanged even after adjusting for several demographic and lifestyle factors. After propensity score matching of 1:2, MetS was also associated with increased risk of bladder cancer (HR = 1.30, 95% CI = 1.06–1.60) (**Table S1**). In the subgroup analyses, MetS-associated risk of bladder did not differ by sex, age, drinking and smoking status, physical activity, and portions of fruit and vegetable intake. We did not find sufficient evidence of interaction effects among pre-specified subgroup factors (**Table S2**).

**Table 2:** Risk of bladder cancer according to metabolic syndrome.

	Cases	Person-years	Incidence rate^*^	HR (95% CI)		
				Model 1	Model 2	Model 3
**Presence of MetS**						
** No**	314	2,685,536	11.7	1.00 (Reference)	1.00 (Reference)	1.00 (Reference)
** Yes**	173	858,571	20.1	1.39 (1.15,1.67)	1.31 (1.08,1.59)	1.32 (1.08,1.61)
**No. of MetS components**						
** 0**	26	490,626	5.3	1.00 (Reference)	1.00 (Reference)	1.00 (Reference)
** 1**	87	1,005,046	8.7	0.9 (0.58,1.4)	0.88 (0.56,1.38)	0.88 (0.56,1.39)
** 2**	146	948,165	15.3	1.23 (0.8,1.87)	1.15 (0.74,1.77)	1.18 (0.76,1.82)
** 3**	123	655,837	18.8	1.37 (0.89,2.11)	1.27 (0.82,1.98)	1.33 (0.85,2.07)
** 4**	79	337,688	23.4	1.68 (1.07,2.63)	1.55 (0.97,2.46)	1.62 (1.01,2.6)
** 5**	26	106,744	24.4	1.7 (0.98,2.95)	1.55 (0.88,2.73)	1.64 (0.92,2.91)
				*p* trend <0.001	*p* trend <0.001	*p* trend <0.001

HR, hazard ratio; CI, confidence interval; MetS, metabolic syndrome.^*^ Per 100,000 person-years.

Model 1: basic Cox proportional hazards model stratified by age, gender and UK Biobank assessment center.

Multivariable adjusted model 2: additionally adjusted for race (white or other), education (college or university degree, other), index of multiple deprivation (a measure of socioeconomic status), smoking status (never smoked, previous smoker, current smoker), alcohol consumption (never or special occasions only, one to three times a month, one to four times a week, daily or almost daily), physical activity (MET hours/week, continuous), portions of fruit and vegetable intake (<5 portions per day, ≥5 portions per day, or unknown/missing).

Multivariable adjusted model 3: fully adjusted model additionally adjusted for regular medications [multivitamin use (yes or no), mineral supplement (yes or no), non-steroidal anti-inflammatory drugs (yes or no), aspirin (yes or no)], and general health indicator variables, including overall health rating (poor, fair, good, excellent, unknown/missing) and long-standing illness (yes or no).

The results showed that the risk of bladder cancer increased with an increasing number of MetS components (*p* < 0.01) (**Table 2**). Among the MetS components, central obesity (HR = 1.39, 95% CI = 1.15–1.68), dyslipidemia for HDL cholesterol (HR = 1.31, 95% CI = 1.04–1.66), and hyperglycemia (HR = 1.44, 95% CI = 1.16–1.79) were all positively associated with bladder cancer risk (*p* < 0.01) (**Table 3**).

**Table 3:** Risk of bladder cancer according to each component of metabolic syndrome.

	Cases	Person-years	Incidence rate^*^	HR (95% CI) ^#^		
				Model 1	Model 2	Model 3
**Central obesity**						
** No**	257	2,250,912	11.4	1.00 (Reference)	1.00 (Reference)	1.00 (Reference)
** Yes**	224	1,283,119	17.5	1.46 (1.22,1.75)	1.38 (1.14,1.66)	1.39 (1.15,1.68)
**Dyslipidemia for triglycerides**						
** No**	155	1,711,727	9.1	1.00 (Reference)	1.00 (Reference)	1.00 (Reference)
** Yes**	299	1,635,967	18.3	1.2 (0.98,1.46)	1.13 (0.92,1.39)	1.14 (0.92,1.4)
**Dyslipidemia for HDL cholesterol**						
** No**	299	2,388,301	12.5	1.00 (Reference)	1.00 (Reference)	1.00 (Reference)
** Yes**	105	645,213	16.3	1.36 (1.09,1.7)	1.29 (1.03,1.63)	1.31 (1.04,1.66)
**Hypertension**						
** No**	60	965,173	6.2	1.00 (Reference)	1.00 (Reference)	1.00 (Reference)
** Yes**	426	2,575,247	16.5	1.27 (0.96,1.68)	1.28 (0.96,1.7)	1.28 (0.97,1.71)
**Hyperglycemia**						
** No**	280	2,464,964	11.4	1.00 (Reference)	1.00 (Reference)	1.00 (Reference)
** Yes**	138	613,814	22.5	1.42 (1.16,1.75)	1.41 (1.14,1.74)	1.44 (1.16,1.79)

HR, hazard ratio; CI, confidence interval; HDL, high-density lipoprotein. ^*^Per 100,000 person-years.

^#^Estimated effects were based on the fully adjusted model (see the footnote in **Table 2**).

**Figure 1:**
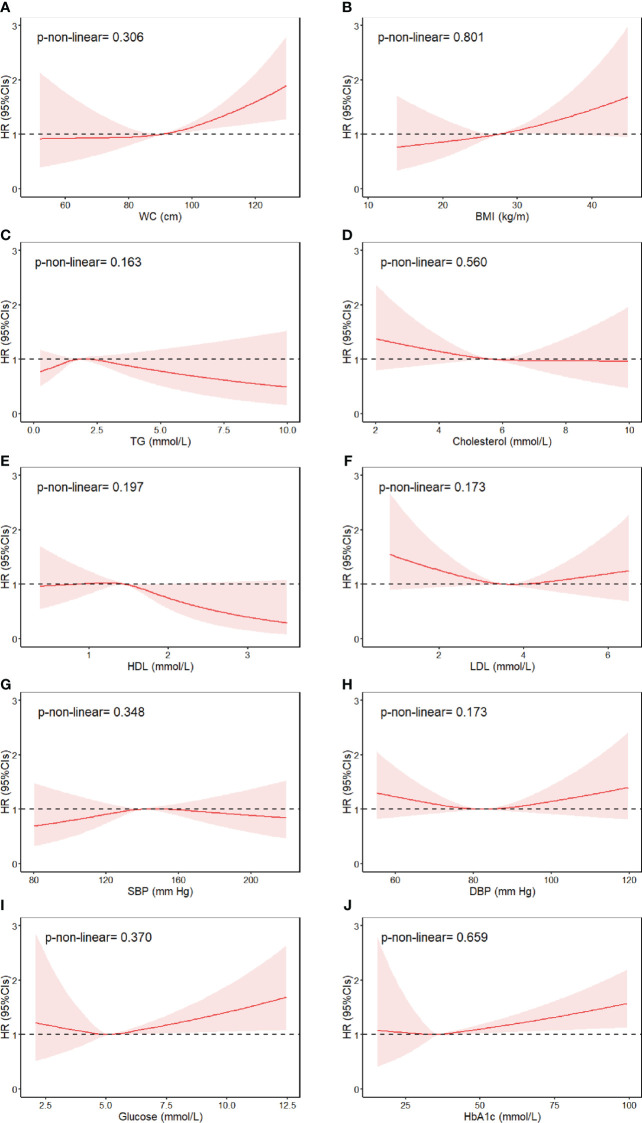
Association between individual MetS components and risk of bladder cancer, allowing for non-linear effects. **(A)** WC, **(B)** BMI, **(C)** TG, **(D)** Cholesterol, **(E)** HDL, **(F)** LDL, **(G)** SBP, **(H)** DBP, **(I)** Glucose, **(J)** HbA1c. The reference levels of MetS components in these plots (with HR fixed as 1.0) were 90.27 cm for WC, 27.42 kg/m^2^ for BMI, 1.74 mmol/L for triglyceride, 5.69 mmol/L for cholesterol, 1.45 mmol/L for HDL-cholesterol, 3.56 mmol/L for LDL-cholesterol, 139.6 mm Hg for SBP and 82.25 mm Hg for DBP, 5.12 mmol/L for fasting glucose, and 36.08 mmol/L for HbA1c. Separate models were fitted using restricted cubic spline with three knots at 10th, 50th, and 90th percentiles for each MetS component, adjusted for age at baseline, gender, UK Biobank assessment center, race, education, index of multiple deprivation, smoking status, alcohol consumption, physical activity, portions of fruit and vegetable intake, regular medications (multivitamin use, mineral supplement, non-steroidal anti-inflammatory drugs, and aspirin), and general health indicator variables (overall health rating and long-standing illness).

We further evaluated the non-linear associations of continuous individual MetS components and bladder cancer risk. Results showed that higher waist circumference, BMI, fasting glucose, and HbA1c were associated with substantially increased risk of bladder cancer, with no evidence against non-linearity (**Figure 1**). Specifically, per unit of increase in waist circumference (1 cm), BMI (1 kg/m^2^), fasting glucose (1 mmol/L), and HbA1c (1 mmol/L), the risk of bladder cancer increased by 2%, 3%, 5%, and 1%, respectively (**Table 4**). There was no sufficient evidence of associations between other MetS components and risk of bladder cancer.

**Table 4:** Risk of bladder cancer in the UK biobank cohort in relation to individual MetS components.

MetS components	HR (95% CI) ^#^		
	Model 1	Model 2	Model 3
**Waist circumference, cm**	1.02 (1.01,1.02)	1.01 (1.00,1.02)	1.01 (1.00,1.02)
**BMI, kg/m^2^**	1.03 (1.01,1.05)	1.03 (1.01,1.05)	1.03 (1.00,1.05)
**Triglycerides, mmol/L**	1.01 (0.93,1.10)	0.98 (0.90,1.07)	0.98 (0.90,1.07)
**Cholesterol, mmol/L**	0.97 (0.89,1.05)	0.96 (0.88,1.04)	0.96 (0.87,1.04)
**HDL, mmol/L**	0.76 (0.56,1.02)	0.8 (0.59,1.09)	0.8 (0.59,1.10)
**LDL, mmol/L**	0.97 (0.87,1.08)	0.96 (0.86,1.07)	0.96 (0.86,1.08)
**SBP, mmHg**	1.00 (1.00,1.00)	1.00 (1.00,1.00)	1.00 (1.00,1.00)
**DBP, mmHg**	1.00 (0.99,1.01)	1.00(0.99,1.01)	1.00 (0.99,1.01)
**Fasting glucose, mmol/L**	1.05 (1.00,1.12)	1.06 (1.00,1.12)	1.05 (1.00,1.12)
**HbA1c, mmol/L**	1.01 (1.00,1.02)	1.01 (1.00,1.01)	1.01 (1.00,1.01)

BMI, body mass index; HDL, high-density lipoprotein; LDL, low-density lipoprotein; SBP, systolic blood pressure; DBP, diastolic blood pressure.

^#^Estimated effects were based on the fully adjusted model (see the footnote in **Table 2**).

## Discussion

In this study, we performed a prospective cohort study involving 476,986 participants from the UK Biobank data set, with almost 500 incident bladder cancer cases. The results showed that MetS (HR = 1.32, 95% CI = 1.08–1.61) and three components, namely, central obesity (HR = 1.39, 95% CI = 1.15–1.68), dyslipidemia for HDL cholesterol (HR = 1.31, 95% CI = 1.04–1.66), and hyperglycemia (HR = 1.44, 95% CI = 1.16–1.79), were associated with increased risk of bladder cancer. We did not find sufficient evidence of non-linear associations between MetS components and risk of bladder cancer.

Recent epidemiological analyses showed that the current incident rate of MetS is approximately 15.5% in China, 35% in the United States (35), and about 30% in Middle Eastern countries (36). More and more studies have shown that MetS is a risk factor for various types of cancers (37–39) and have increasingly aroused general concern. Our study suggested that MetS increased by 32% the incident risk of bladder cancer, which is consistent with a previous study (13). Additionally, a prospective cohort study conducted by Haggstrom (40) and a meta-analysis conducted by Esposito (17) showed that, in men, MetS was associated with increased risk of bladder cancer, but not in women. Nevertheless, our findings confirmed the association with moderate HR in both men and women. The discrepancy between previous studies and our study might be caused by racial difference among participants, different definitions of MetS, and sample size.

Each component of MetS was reported to be associated with cancer development, but it remains unclear whether the effects of these components are additive or synergistic. In this study, the risk of bladder cancer was evaluated by associating it with overweight and dyslipidemia for HDL cholesterol. Studies have shown that the increase in free fatty acid (FFA) and abnormal adipokines caused by obesity can lead to insulin resistance and may lead to the onset of MetS (41, 42). Obesity, especially visceral obesity, leads to chronic low-grade inflammation of the whole body, which is attributed to the production of inflammatory cytokines by fat cells and infiltrating immune cells, forming a carcinogenic environment (43, 44). Previous studies reported inconsistent results on BMI and bladder cancer risk, some studies have shown a positive association (45, 46), and several studies reported no significant correlation (47, 48). Our study showed that higher BMI and central obesity were positively associated with bladder cancer risk.

Hyperglycemia was also found to be a risk factor in this study. Hyperglycemia may affect the risk of cancer and cancer-related death through the direct mitogenic effect of insulin and the production of IGF-1 (insulin-like growth factor 1) (49, 50). IGF-1 affects cell growth, abnormal proliferation, and differentiation through endocrine, paracrine, and autocrine mechanisms (51). Insulin resistance is also essential for the development of hyperglycemia-induced tissue damage, hypertension, and dyslipidemia (52, 53). In addition, the incidence of urinary tract infections in diabetic patients increases and the long-term chronic urinary tract inflammation may stimulate the malignant transformation of the urothelium (54, 55). Some cohort studies have addressed the positive association between high blood glucose, diabetes, and bladder cancer, but only some of these studies have a significant odds ratio (56–58).

For hypertension, no consensus was met on its relationship with cancer, including bladder cancer risk. A case–control study demonstrated that untreated hypertension was associated with a reduced risk of bladder cancer (59). Oppositely, we found an increasing trend, but the result did not reach statistical significance. In other studies, no association was shown between them (60, 61). Hypertension also showed a controversial role in pathological staging and grading (62, 63). Consequently, its impact on the occurrence and development of bladder cancer and related pathways needs to be further studied.

A key strength of our research is that the data were based on a nationwide prospective database, with a large sample size and detailed measurements. This allowed for simultaneous adjustment for widely known and putative risk factors in the associations of interest. In addition, we assessed the linear and non-linear relationship between individual components of MetS and bladder cancer, which has rarely been revealed in existing epidemiological studies.

Our research has some limitations. First, as an observational study, the causality between MetS and bladder cancer could not be confirmed. Second, as with all observational studies, residual confounding remains possible. Third, analysis of the effects of MetS on bladder cancer subtypes is limited due to the lack of histological information. Fourth, the “healthy volunteer” selection bias of the UK Biobank might limit the generalization of our findings (64). Last, since the MetS was defined only once based on MetS components measurements collected at enrollment during 2006–2010 in the UK Biobank, the effect of alterations in these risk factors over time could not be assessed.

## Conclusion

Overall, the present study suggested that MetS might be associated with increased risk of bladder cancer in the general population. Central obesity, dyslipidemia for HDL cholesterol, and hyperglycemia were the three predominant MetS components that might be independently associated with the risk of bladder cancer. The primary associations were weak and may be influenced by residual confounding. Future larger prospective studies are needed to confirm our conclusion and investigate the external validity of our findings.

## Data availability statement

The data used in this study is available under license from the UK Biobank (application number 51671, approved August 2019) but restrictions apply. Further inquiries can be directed to the corresponding authors.

## Ethics statement

The study was approved by the North West Multi-center Research Ethics Committee, the England and Wales Patient Information Advisory Group, and the Scottish Community Health Index Advisory Group (application number 51671, approved August 2019). Before collecting data, all participants provided written informed consent.

## Author contributions

SF, JY, BX, and YP designed the study. TG, LZ, and ZZ obtained and assembled the data. RS and JY analyzed and interpreted the data. SF, YL, and HD wrote the paper. BX and YP revised the manuscript. All authors reviewed the manuscript and approved the final version.

## Funding

This research was supported by grants including (1) Guangdong Provincial Natural Science Fund project (2021A1515010807); (2) Guangdong Basic and Applied Basic Research Regional Combination The Youth Foundation (2019A1515110155); (3) Shenzhen Natural Science Foundation Basic Research Surface Project (JCYJ20210324123012035); 4) the Fundamental Research Funds for the Central Universities, Sun Yat-sen University (Grant No. 22qntd3702); and 5) National Natural Science Foundation of China (NSFC) Youth Science Fund Project (32200583).

## Acknowledgments

This work was conducted using the UK Biobank Resource (application number 51671). We thank the participants and staff of the UK Biobank cohort for their valuable contributions.

## Conflict of interest

The authors declare that the research was conducted in the absence of any commercial or financial relationships that could be construed as a potential conflict of interest.

## Publisher’s note

All claims expressed in this article are solely those of the authors and do not necessarily represent those of their affiliated organizations, or those of the publisher, the editors and the reviewers. Any product that may be evaluated in this article, or claim that may be made by its manufacturer, is not guaranteed or endorsed by the publisher.

## Abbreviations

BMI, body mass index; CI, confidence interval; HDL, high-density lipoprotein; HR, hazard ratio; IDF, International Diabetes Federation; IGF-1, insulin-like growth factor 1; LDL, low-density lipoprotein; MetS, metabolic syndrome; SD, standard deviation; TG, triglyceride; WC, waist circumference.
