# Supplementary material for: Association of metabolic syndrome and the risk of bladder cancer: A prospective cohort study
Source: Front Oncol. 2022 Oct 3;12:996440. doi: 10.3389/fonc.2022.996440 (PMC9574437; doi:10.3389/fonc.2022.996440)
Supplement: Supplementary file 1 [file Table_1.docx]

**Table S1. HRs (95% CI) for the association between MetS and risk of bladder cancer using propensity score–matching approach in the UK biobank.**

|  | **Non-MetS** (N=194,345) |  | **MetS**  (N= 111,437) |
| --- | --- | --- | --- |
| **Cases** | 294 |  | 216 |
| **Person-years** | 1,439,052 |  | 822,179 |
| **Incidence rate^*^** | 20.4 |  | 26.3 |
| **HR (95% CI)** | 1.00 (Ref) |  | 1.30 (1.06, 1.60) |

Abbreviations: CI, confidence interval; HR, hazard ratio, **MetS**, metabolic syndrome.

^*^ Per 100,000 person years.
