# Supplementary material for: Association of metabolic syndrome and the risk of bladder cancer: A prospective cohort study
Source: Front Oncol. 2022 Oct 3;12:996440. doi: 10.3389/fonc.2022.996440 (PMC9574437; doi:10.3389/fonc.2022.996440)
Supplement: Supplementary file 2 [file Table_2.docx]

**Table S2. Stratified analyses of metabolic syndrome and risk of bladder cancer**

|  | **Cases** | **Person-years** | **Incidence rate** | **Multivariate-adjusted HR (95%CI)** | ***P* interaction** |
| --- | --- | --- | --- | --- | --- |
| **Sex** |  |  |  |  |  |
| **Male** | 105 | 1,927,813 | 0.54 | 1.25 (0.99,1.56) |  |
| **Female** | 382 | 1,620,041 | 2.36 | 1.56 (1.02,2.4) | 0.191 |
| **Age** |  |  |  |  |  |
| **<60** | 115 | 2,083,705 | 0.55 | 1.46 (0.96,2.22) |  |
| **≥60** | 372 | 1,464,149 | 2.54 | 1.29 (1.03,1.61) | 0.589 |
| **Never smoker** |  |  |  |  |  |
| **Yes** | 151 | 1,970,268 | 0.77 | 1.55 (1.07,2.25) |  |
| **No** | 336 | 1,577,586 | 2.13 | 1.19 (0.94,1.51) | 0.672 |
| **Never drinker** |  |  |  |  |  |
| **Yes** | 26 | 292,583 | 0.89 | 1.21 (0.49,3.02) |  |
| **No** | 461 | 3,255,271 | 1.42 | 1.34 (1.09,1.64) | 0.448 |
| **Physical activity** |  |  |  |  |  |
| **< median** | 295 | 2,134,731 | 1.38 | 1.37 (1.07,1.77) |  |
| **≥median** | 192 | 1,413,122 | 1.36 | 1.25 (0.9,1.72) | 0.777 |
| **Below 5 portions of fruit and vegetable per day** |  |  |  |  |  |
| **Yes** | 319 | 2,211,124 | 1.44 | 1.27 (0.99,1.62) |  |
| **No** | 168 | 1,336,729 | 3.47 | 1.43 (1.02,2.02) | 0.388 |
| Estimated effects were based on the fully adjusted model (see the footnote in Table 2) | | | | | |
